# Impact of Feed Delivery Pattern on Aerial Particulate Matter and Behavior of Feedlot Cattle †

**DOI:** 10.3390/ani7030014

**Published:** 2017-03-01

**Authors:** Frank M. Mitloehner, Jeff W. Dailey, Julie L. Morrow, John J. McGlone

**Affiliations:** 1 Department of Animal and Food Sciences, Texas Tech University, Lubbock, TX 79409, USA; john.mcglone@ttu.edu; 2 Livestock Issues Research Unit, USDA-ARS, Lubbock, TX 79409, USA; jefftadailey@gmail.com (J.W.D.); fmmitloehner@ucdavis.edu (J.L.M.)

**Keywords:** behavior, feeding management, feedlot cattle, particulate matter

## Abstract

**Simple Summary:**

Fine particulate matter (with less than 2.5 microns diameter; aka PM_2.5_) are a human and animal health concern because they can carry microbes and chemicals into the lungs. Particulate matter (PM) in general emitted from cattle feedlots can reach high concentrations. When feedlot cattle were given an altered feeding schedule (ALT) that more closely reflected their biological feeding times compared with conventional morning feeding (CON), PM_2.5_ generation at peak times was substantially lowered. Average daily generation of PM_2.5_ was decreased by 37% when cattle behavior was redirected away from PM-generating behaviors and toward evening feeding behaviors. Behavioral problems such as agonistic (i.e., aggressive) and bulling (i.e., mounting each other) behaviors also were reduced several fold among ALT compared with CON cattle. Intake of feed was less and daily body weight gain tended to be less with the altered feeding schedule while efficiency of feed utilization was not affected. Although ALT may pose a challenge in feed delivery and labor scheduling, cattle had fewer behavioral problems and reduced PM_2.5_ generation when feed delivery times matched with the natural drive to eat in a crepuscular pattern.

**Abstract:**

Fine particulate matter with less than 2.5 microns diameter (PM_2.5_) generated by cattle in feedlots is an environmental pollutant and a potential human and animal health issue. The objective of this study was to determine if a feeding schedule affects cattle behaviors that promote PM_2.5_ in a commercial feedlot. The study used 2813 crossbred steers housed in 14 adjacent pens at a large-scale commercial West Texas feedlot. Treatments were conventional feeding at 0700, 1000, and 1200 (CON) or feeding at 0700, 1000, and 1830 (ALT), the latter feeding time coincided with dusk. A mobile behavior lab was used to quantify behaviors of steers that were associated with generation of PM_2.5_ (e.g., fighting, mounting of peers, and increased locomotion). PM_2.5_ samplers measured respirable particles with a mass median diameter ≤2.5 μm (PM_2.5_) every 15 min over a period of 7 d in April and May. Simultaneously, the ambient temperature, humidity, wind speed and direction, precipitation, air pressure, and solar radiation were measured with a weather station. Elevated downwind PM_2.5_ concentrations were measured at dusk, when cattle that were fed according to the ALT vs. the CON feeding schedule, demonstrated less PM_2.5_-generating behaviors (*p* < 0.05). At dusk, steers on ALT vs. CON feeding schedules ate or were waiting to eat (standing in second row behind feeding cattle) at much greater rates (*p* < 0.05). Upwind PM_2.5_ concentrations were similar between the treatments. Downwind PM_2.5_ concentrations averaged over 24 h were lower from ALT compared with CON pens (0.072 vs. 0.115 mg/m^3^, *p* < 0.01). However, dry matter intake (DMI) was less (*p* < 0.05), and average daily gain (ADG) tended to be less (*p* < 0.1) in cattle that were fed according to the ALT vs. the CON feeding schedules, whereas feed efficiency (aka gain to feed, G:F) was not affected. Although ALT feeding may pose a challenge in feed delivery and labor scheduling, cattle exhibited fewer PM_2.5_-generating behaviors and reduced generation of PM_2.5_ when feed delivery times matched the natural desires of cattle to eat in a crepuscular pattern.

## 1. Introduction

A small portion of the particulate matter (PM) released under commercial feedlot conditions is considered fine. However, particulate matter smaller than 2.5 μm in diameter (PM_2.5_) is considered a health hazard to humans and animals because it can pass through the respiratory tract to the lung alveoli [1,2,3] and therefore of major interest. These fine particles can carry gases and microbes into the lung [4].

Exposure to fine PM causes increased respiratory and cardiovascular health issues [5,6,7] and the biological response of the respiratory system varies based on particle size [8]. Fine particulate matter such as PM_2.5_, more likely enters the lungs and studies have shown that components of PM, such as endotoxin or pathogens, can lead to cellular inflammation [9].

Particulate matter generation around cattle feedlots is greater in the evening compared with the morning because of increased cattle activity and is usually correlated with dry weather events [10]. Behavioral patterns of feedlot cattle differ from those displayed on pastures. Cattle kept in a pasture setting show a crepuscular pattern of activity [11,12], expressing feeding behaviors that peak (i.e., show increase in feeding behavior) at dusk and dawn. They also express less feeding behavior mid-day. In commercial feedlots, cattle are often fed during the first half of the daylight hours to maximize labor utilization and lower production costs. However, this method of feeding does not follow the natural behavior that cattle show in the pasture setting. Feed may not be available at dusk when pastured cattle naturally graze [12].

We hypothesized that by changing the feeding times of feedlot cattle from morning feeding to a more crepuscular feeding rhythm (i.e., feeding at dusk and dawn), PM_2.5_-generating behaviors could be redirected into feeding and thereby decrease aerial particulate matter concentrations. The objectives of this study were to (1) determine cattle behaviors associated with PM_2.5_ generation; and (2) determine if feeding in the evening can alter cattle behaviors during periods of PM_2.5_ generation and lower generated particulate matter.

## 2. Materials and Methods

### 2.1. General

An experiment was conducted in a large-scale (>60,000 cattle) commercial feedlot in west Texas in order to examine the effect of altering feeding patterns on cattle behavior and resulting particulate matter generation. The experiment was conducted from April through May 2000, and was approved by the Texas Tech University Animal Care and Use Committee (IACUC#2000-02).

This experiment compared 2 feeding regimens: normal-scheduled feeding (CON) at 0700, 1000, and 1200 and alternative-scheduled feeding (ALT) at 0700, 1000 and 1830. Alternative-scheduled feeding was developed to mimic crepuscular feeding times that are normally found in grazing cattle. For CON, daily ration was fed in 3 equal portions at each scheduled time. For ALT, 30%, 20%, and 50% of the daily ration was fed at 0700, 1000, and 1830, respectively. Drinking water was provided for *ad libitum* intake.

The cattle in both treatments were fed using a clean bunk management system. In this system, the goal was to have no feed remaining in the feed bunk for a few hours before the first feeding in the morning. To ensure that cattle are eating at approximately *ad libitum* rates, feed deliveries were typically held constant for a 3-day period, after which the cattle were “challenged” to consume more feed by increasing the feed delivery by approximately 0.1 kg/animal daily (as-fed basis). If the feed bunk was empty at the desired time before the morning feeding for a period of 3 days after the “challenge,” the challenge was repeated; if not, the intake was decreased to the level before the challenge.

### 2.2. Pens and Cattle

**Figure 1:**
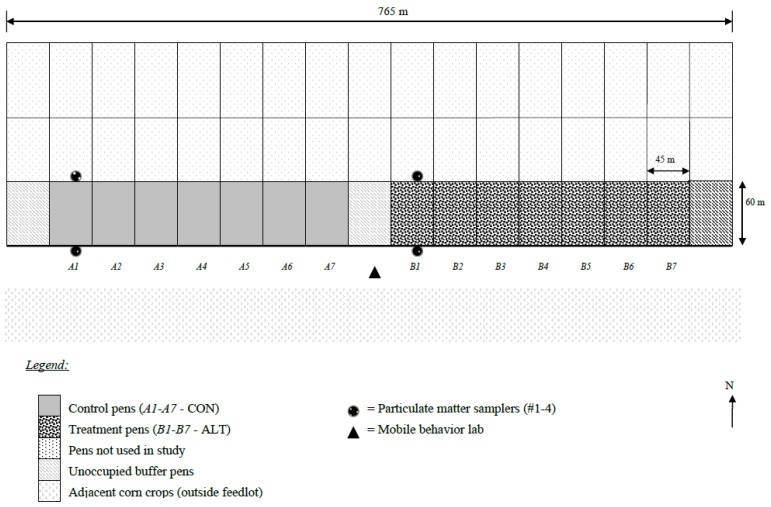
Schematic of experimental setup for particulate matter (PM_2.5_) and behavior sampling. A total of 4 PM_2.5_ samplers were used to measure PM_2.5_ concentrations upwind (south side) and downwind (north side) in the south and southwest pens of the feedlot, in 1 pen per treatment per day. The 4 PM_2.5_ samplers were moved daily from 1 of the 7 pens (per treatment) to the next. For example, on d 1, upwind and downwind locations were sampled in pens A1 and B1 as shown above. On day 2, upwind and downwind locations were sampled for PM_2.5_ in pens A2 and B2 etc. Behavioral measures were recorded in 15 min scan sampling mode by live observation using a camera that was mounted to a 10-m high rotating tripod on top of a mobile behavior lab from 1600 and 2100 on the same days when PM_2.5_ was measured. The mobile behavior lab remained at the depicted location throughout the duration of the study. Prevailing wind direction was from the South.

Figure 1 shows a schematic of 14 experimental pens (7 CON, 7 ALT, separated by one unoccupied buffer pen) with east-west orientation located at the south end of the feedlot (adjacent to a crop field). Because south winds predominated, this orientation minimized cross contamination of PM_2.5_ between experimental corrals and the other corrals in the feedlot that were not part of the study. It was a practical necessity to apply the treatments in groups of pens (7 ALT and 7 CON) because animal behaviors are affected by cattle in adjacent pens. Feeding schedule (i.e., delivery times) had be grouped in spatially adjacent pens because otherwise, behavior of cattle in CON pens would have been affected by the cattle feeding activity in the ALT cattle and vice versa.

One pen on each side of the ALT and CON corrals was left unoccupied to avoid cross-contamination of PM_2.5_ and to limit the above mentioned possible crossover effects of feeding times on behavior.

Pen surfaces (dirt lot) were not shaded and manure was removed from all 14 experimental pens before the trial began.

A total of 2813 crossbred steers, housed in 14 corral pens, were included in the study. The average number of steers per pen was 176 (15.2 m^2^/steer) and with 24 cm of bunk space per steer. Before trial initiation, cattle were implanted with Synovex-S (Fort Dodge Animal Health, Overland Park, KS, USA) or Ralgro (Shering Plough, Kenilworth, NJ, USA). After receiving, cattle were weighed, blocked by arrival time, and randomly assignment to pens within blocks (paired pens) (see Figure 1).

Cattle were weighed at the onset and the conclusion of the experiment to assess BW changes. Average initial BW of steers in ALT vs. CON pens were similar (354.4 vs. 355.2 kg, SE = 8.4 kg). Both delivered and refused feed were recorded and DM assessed to allow for determination of DMI.

Steers in both treatments were adjusted to a finishing diet (Table 1) after approximately 14 days using four adaptation diets (i.e., stepped up in concentrate feed until reaching finishing feedlot ration. Measurements began 1 month after the cattle arrived at the feedlot to allow for adaptation to the environment and feeding schedules.

### 2.3. Equipment

Automated DustTrak PM_2.5_ samplers (Particulate Matter DustTrak, Model 8520, TSI, St. Paul, MN, USA) were used to measure PM_2.5_ concentration. DustTrak samplers are not considered compliant with the Federal Reference Method for measuring PM_2.5_, thus, the results may not accurately present absolute values usable for e.g., regulatory purposes, but rather data are useful to discover relative differences between treatments.

Before use, PM_2.5_ samplers were factory calibrated and cleaned following the manufacturer’s recommended schedule. Additionally, prior to sampling in the field, PM_2.5_ samplers were compared site-by-site to rule out instrument differences as well as any kind of malfunctioning.

PM_2.5_ samplers were automated and based on light scattering technology, which remotely measure PM concentration every minute and log at 15 min intervals. PM_2.5_ samplers measured respirable particles with a mass median diameter ≤2.5 μm (PM_2.5_) every 15 min over a period of 7 days in April and May. In short, the sampler draws air into a sensing chamber, which then is illuminated by a laser beam. The particles scatter light in all directions and a lens concentrates that light on a photodetector, which converts it into voltage. The scattered light is proportional to the voltage and the mass concentration of the aerosol.

A total of 4 samplers were used to measure PM_2.5_ concentrations upwind and downwind in the south and southwest pens of the feedlot, in 1 pen per treatment per day. The 4 PM_2.5_ samplers were moved daily from 1 of the 7 pens (per treatment) to the next. The measured particulate matter size was ≤2.5 μm. PM_2.5_ data were collected on days when the wind was entirely from the south, southeast, or southwest wind direction to prevent cross contamination between treatments.

In addition, there was an unoccupied pen between the seven treatment and seven control pens. The instruments were housed in factory provided weatherproof environmental enclosures, which were placed inside steel cages to protect them from weather and the animals. Cages were attached to the middle of the upwind and downwind corral fence line. PM_2.5_-sampler inlets were placed about 1.50 m above the ground (approximate height of steer heads when standing).

A weather station (Metos, Weiz, Austria) co-located by the mobile behavior lab, measured weather parameters (precipitation, temperature, humidity, wind speed/-direction, air pressure and light intensity) in 10 min periods over the entire study period. All weather sensors were operated at a 2 m height.

### 2.4. Behavior

Behaviors were measured during the periods in which PM_2.5_ was generated (from 1600 until 2100) using a mobile behavior lab. Behavioral measures were recorded by live observation using a camera that was mounted to a 10 m high rotating tripod on top of a mobile behavior lab from 1600 until 2100 over 7 days. After 2100 h, during darkness, observers could not accurately record cattle behaviors. Behavioral data were measured using a 15-min scan sampling technique, in which the total number of cattle (per pen) performing a given behavior, was directly entered into a computer spreadsheet [13]. Data were expressed as a percentage of time of total observations during the period of particulate matter generation. Thus, behavioral measures are not indicative of an average of 24 h/day, but of the targeted period of PM_2.5_ generation (in the evening). Percentage data were analyzed as Least Squares Means (LSM), Standard Error Means (SEM), and *p*-values determined by arcsine square root transforming percent data (to achieve normalized distribution).

The measured behaviors were: (a) PM_2.5_-generating behaviors including locomotion (walk/run), agonistic and bulling behavior; and (b) behaviors with little effect on PM_2.5_ like feeding, waiting for feed, lying and standing behavior. Behaviors were defined as described earlier [13], but in short: Locomotion was any change of body location within the pen. Agonistic behaviors were those indicative of social conflict such as threat, attack, fight, or escape. Bulling behavior was defined as mounting of a steer by its peer(s). Feeding was defined to be head over or in the bunk. Waiting for feed was an upright body posture near the bunk without actually having access to feed. Steers waiting for feed were normally waiting in the second or third row until their feeding peers were finished. Drinking was defined as the head over or in the water trough. Lying was defined as body contact with the ground and standing was considered to be an inactive upright posture (no locomotion).

### 2.5. Experimental Design and Analysis

Behavioral measures were collected during the period of PM_2.5_ generation (1600 to 2100 h) and represent the percentage of time cattle were engaged in these behaviors during that period. Particulate matter measures were collected over 24 h and averaged for each hour of the day. The pen was the experimental unit (cattle had been randomly assigned to experimental units). Treatment pens selected were determined by location in the feedlot.

The experimental design for behavioral and PM_2.5_ measures was a randomized complete block with paired pens on a given arrival date as the blocks (the assignment to treatments within blocks was random). As mentioned earlier, a total of 14 pens were used (7 pens/treatment). For measures of performance and behavior, the statistical model included treatment, block, and treatment by block interaction effects. The treatment by block effect was the error term. The effects of behaviors on the variation in PM_2.5_ were analyzed with the stepwise multiple regression procedure and correlations of behaviors with PM_2.5_ calculated using SAS (SAS Inst, Inc., Cary, NC, USA) in an attempt to predict aerial dust based on behavior. For PM_2.5_ measures, a repeated measures analysis was employed with 24 time periods per day. The PM_2.5_ model was the same as above, but also included effects of time, and treatment by time, and residual error. Performance data were analyzed as a completely randomized design with 2 treatment groups and 7 pen replications per treatment. Because treatments were determined by physical location of the pens and the predominant wind direction, pens could not be randomly assigned treatments (see earlier discussion). However, cattle were randomly assigned to pen experimental units. Means separation using the predicted difference test was governed by protected levels of significance at the alpha level reported. All analysis was conducted using PROC MIXED in SAS (SAS Inst, Inc., Cary, NC, USA).

Finally, correlation coefficients were calculated to examine relationships between cattle behaviors and the variation in aerial PM_2.5_ using SAS.

## 3. Results

### Effects of Feeding Management on Particulate Matter and Behavior

During the afternoon and early evening period, cattle experiencing the ALT treatment showed differences in most behaviors compared to those in CON (Table 2). Time spent feeding was not affected (*p* = 0.40) by treatment. Whereas CON cattle fed throughout the afternoon, the feed bunks in the corrals of ALT cattle contained no feed in the afternoon hours. Feeding behavior of cattle in the ALT treatment was concentrated after feed was delivered for a period of about 90 min (1830 to 2000). “Waiting for feed” behavior was different between cattle in CON vs. ALT treatments (*p* < 0.001).

Cattle fed according to ALT schedule started lining up at the feed bunks approximately 30 min before feed was delivered. Cattle fed according to CON vs. ALT treatments spent more time drinking (*p* < 0.028). Cattle experiencing CON vs. ALT treatments showed more standing behavior (*p* < 0.01). Lying behavior was not different between treatments. The locomotive behaviors walking and running were performed less among cattle experiencing the ALT than the CON treatments (*p* < 0.004). Agonistic behavior was performed three times less among ALT versus CON treated cattle (*p* < 0.002) and bulling behavior was lower among ALT versus CON treated cattle (*p* < 0.05).

Table 3 shows correlations between behaviors and PM2.5 net concentrations for both treatments. Correlation coefficients were homogenous within treatments which indicates that PM2.5 generation, even if different in mean value in different treatments have similar causal behaviors. In ALT, net PM2.5 and the different behaviors showed significant correlations with standing (*r* = 0.45, *p* < 0.01), lying (*r* = −0.43, *p* < 0.01), and agonistic behaviors (*r* = 0.35, *p* < 0.05). The stepwise multiple regression showed that agonistic behavior predicted 17% of the variation in dust (partial R^2^ = 0.17).

Over the 24 h period (Table 4), upwind PM_2.5_ concentrations were similar between treatments. PM_2.5_ concentrations measured at downwind locations were 37% lower among pens housing ALT compared to CON treated cattle (*p* < 0.01). Net PM_2.5_ concentration was different between treatments; pens housing ALT vs. CON cattle had 55% lower PM_2.5_ particulate matter concentrations (*p* < 0.05).

**Figure 2:**
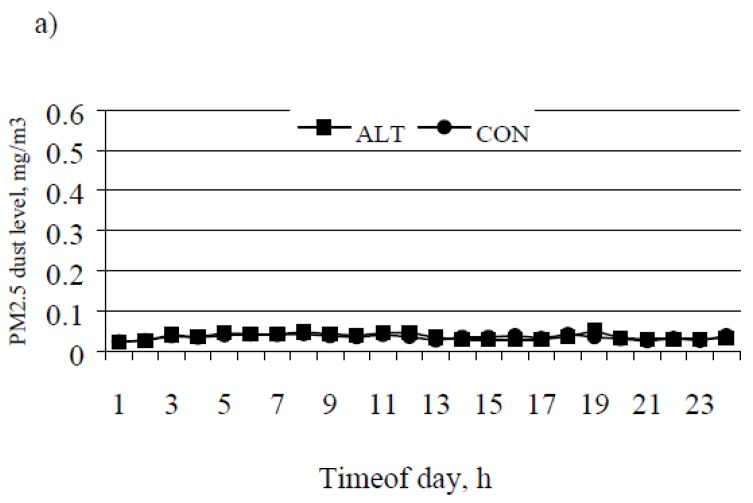

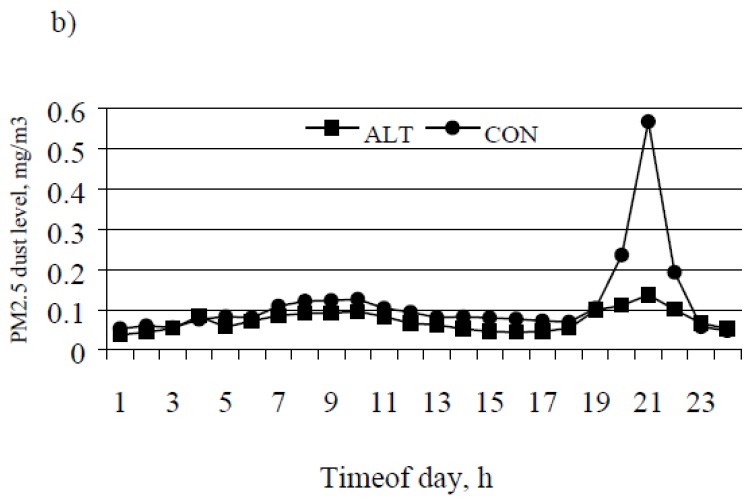
Average PM_2.5_ (particulate matter, <2.5 μm) concentrations over 24 h in mg/m^3^ in a West-Texas feedlot under 2 different feeding time regimens (ALT = fed at 0700, 1000 and 1200 h vs. CON = fed at 0700, 1000 h and 1830 h). PM_2.5_ was measured over a period of 7 days in April 2000. Panel (**a**) shows upwind PM_2.5_ concentration, which was 0.035 mg/m^3^ in ALT versus 0.036 mg/m^3^ in CON (Pooled SE; SEM = 0.006, Treatment *p* = 0.87, Treatment by time *p* = 0.08). Panel (**b**) shows average downwind PM_2.5_ concentrations, which was 0.072 mg/m^3^ in ALT vs. 0.115 mg/m^3^ in CON (SEM = 0.007, Treatment *p* < 0.01, Treatment by time *p* < 0.01).

In Figure 2a, hourly averages of upwind PM_2.5_ concentrations are presented over 24 h. The two treatments had similar upwind PM_2.5_ concentrations. Figure 2b shows the average hourly downwind PM_2.5_ concentrations of the two treatments over 24 h. Between 2000 and 2200, pens housing CON vs. ALT treated cattle had approximately four times higher PM_2.5_ concentrations (*p* < 0.05).

Initial body weights were similar between treatments. The DMI of steers in ALT was 3.6% less (*p* < 0.01) than in CON, which led to a tendency (*p* = 0.095) for a lower ADG. A post-hoc, secondary analysis was performed to determine if DMI was held constant by use of a covariate, if ADG differed between treatments. When DMI was held constant by use of a covariate, then ADG was similar (*p* = 0.88) for cattle in CON and ALT treatments (Table 5).

## 4. Discussion

It is generally accepted that hot and dry weather is conducive to detachment of particles from the feedlot pen surface [14]. The problem of high particulate matter concentrations are most severe during the late afternoon and early evening hours, when the ambient temperatures are highest and the relative humidity lowest. The moisture level of the manure pad in the feedlot pen, vapor pressure in the air, and precipitation undoubtedly affect particulate matter concentrations [14,15]. The cohesion between particles is lowest during dry periods and during those times, detachment of particles from the ground is easiest [16]. However, additional work is needed to clarify these potential relationships.

Other PM work indicated that the high downwind PM concentrations which occur in feedlots in the evening are affected by a strong increase in cattle activity [16,17]. We hypothesized in the present study that the common feeding practice of only feeding cattle in the morning impacts this problem. A change from conventional to more crepuscular feeding times (dusk, noon, and dawn) was proposed to change PM-generating behavior into those that are less active, and therefore generate less particulate matter. Particulate matter-generating behaviors (like agonistic and bulling behaviors) were much lower in the alternative feeding time regime than in the conventional control. Cattle have a strong feeding motivation around dawn and dusk [17], and if feed is unavailable, we hypothesized that cattle will replace feeding with locomotion, agonistic, or bulling behavior.

Correlations between behavior and PM_2.5_ generation do not indicate cause and effect. However, one can see from our data that two behaviors (agonistic behaviors and standing) had a significant (though not perfect) correlation with PM_2.5_ generation (Table 3). These correlations between behavior and fine particle generation support the hypothesis that active behaviors stir up PM_2.5_ while less-active behaviors (e.g., feeding) do not. Lying down is negatively correlated with dust levels because when animals lay down, less PM_2.5_ is generated and when they are not lying down, they may be active fighting or standing which do generate PM_2.5_. A key finding of this work is the concept that cattle behavior contributes to PM_2.5_ generation in a meaningful way. Furthermore, when cattle management/feeding practices are changed to reduce active behaviors, PM_2.5_ generation was being reduced.

The findings of this study were consistent with the hypothesis that active behaviors are associated with PM_2.5_ generation. At dusk, when the majority of agonistic and locomotive behaviors occur, PM_2.5_ concentrations peaked. By altering feeding times, the agonistic behaviors were reduced and consequently PM_2.5_ concentrations were also reduced. Altered feeding schedule clearly caused reduced PM_2.5_ concentrations. Behavioral data supports the hypothesis that increasing feeding behaviors and reducing agonistic behaviors in the evening will reduce PM_2.5_ generation. Further work is needed to quantify the PM distribution generated by cattle feedlots. It would be interesting to know PM_5_ and PM_10_ concentrations in this production system and to see if our new management practice might change the distribution of particles of various sizes.

Conducting in-field management studies on PM_2.5_ movement necessitates accounting for wind direction and has the limitations of the physical location of pens within the feedlot. Adjoining pens/experimental units can influence each other and thus, the experimental units cannot be totally independent. However, because behaviors were different between treatments, it was clear that our management change (altered feeding times) caused changes in cattle behavior and dust generation that were larger than any effects that adjoining pens may have caused.

Future studies should determine the relationships between feeding levels and PM_2.5_ generation. Feeding a high proportion of the daily ration in the early evening might alter the feeding behavior of cattle the next morning, which might affect management decisions on allotment of feed to each pen.

In the USA, PM_2.5_ is regulated by the federal and state agencies and cattle feedlots can run the risk of exceeding the thresholds. Approaches are needed to reduce PM_2.5_ emissions from confined cattle feeding operations. The present research may suggest a management practice that may reduce PM_2.5_ emissions from confined animal feeding operations. While this work was conducted in a commercial environment, more research is needed to refine this approach, independently confirm its efficacy, and understand how this approach may impact labor efficiency and profitability.

## 5. Conclusions

In summary, when the feeding of cattle in a feedlot is managed to reflect biological and behavioral motivations for feed consumption, behaviors that affect the generation of PM_2.5_ were decreased. The study showed that cattle behaviors are an important factor and perhaps the main reason for high PM_2.5_ levels in the evenings. Use of an altered schedule like the one described herein may decrease the generation of PM_2.5_ by altering cattle behavior. Use of a behavioral management schedule that is more consistent with the natural cattle feeding cycle might improve human and animal health and well-being.

### Implications

Crepuscular cattle feeding patterns have led to significantly lower PM_2.5_ concentrations compared to common feeding practices (0700, 1000, 1200 h). By changing cattle feeding patterns, a producer may be able to redirect PM_2.5_ generating behaviors to more environmentally benign behaviors, thereby lowering PM_2.5_ concentrations in feedlots.

## Figures and Tables

**Table 1:** Diet fed to cattle in a West Texas feedlot.

Feed Ingredients	% DM in Diet
Flaked milo	44.14
Corn silage	15.74
Supplement premix	3.33
Fat	2.59
Liquid premix	1.43
Water	0.48
Milo screen	0.34
Nutrients, %	
DM	68.04
CP	13.80
Fat	8.00
CF	4.73
Ca	0.70
P	0.30
K	0.70
Mg	0.22
Salt	0.30
S	0.24
NEm	220.76
NEg	144.32

Notes: The feed additives Tylan (7.4 mg/kg) and Rumensin (20.9 mg/kg) were added to the diet (Elanco Animal Health, Indianapolis, IN, USA) and fed throughout the trial.

**Table 2:** Behaviors of cattle (% of time) under 2 different feeding-time regimens (ALT = fed at 0700, 1000, 1830 h vs. CON = fed at 0700, 1000 and 1200 h) measured for 7 d from 1600 until 2100.

Behavior	Alternative Feeding	Control Feeding	SEM ^a^	*p*-Value
Number of Replicates	7	7		
Number of Animals	1228	1585		
Feeding	11.0	6.5	0.80	0.40
Waiting for feed	19.3	4.6	1.57	0.001
Drinking	2.2	2.4	0.07	0.028
Standing	31.0	54.3	2.43	0.001
Lying	34.5	26.9	2.61.	0.20
Walking	1.6	2.8	0.21	0.004
Agonistic behavior	0.8	2.4	0.20	0.002
Bulling	0.003	0.013	0.003	0.050

**^a^** SEM = Standard error means.

**Table 3:** Correlations of behaviors of cattle (% of time over 24 h) with PM_2.5_ net concentration under two feeding-time regimens (ALT = fed at 0700, 1000, 1830 h vs. CON = fed at 0700, 1000 and 1200 h) measured for 7 days from 1600 until 2100.

Behavior	PM-2.5 Net Concentration	
	Alternative Feeding	Control Feeding
*Feeding*	0.22	−0.10
*Waiting for feed*	0.17	0.11
*Drinking*	−0.13	−0.29
*Standing*	0.45 *	0.34 *
*Lying*	−0.43 *	−0.34 *
*Locomotion*	0.24	0.32 ^†^
*Agonistic*	0.44 **	0.35 *
*Bulling*	0.16	0.26

* *p* < 0.05. ** *p* < 0.01. ^†^ *p* < 0.06.

**Table 4:** Average particulate matter concentrations (particle size < 2.5 μm, PM_2.5_) in a West Texas feedlot under 2 different feeding time regimes (ALT = 0700, 1000, 1830 h vs. CON = 0700, 1000, and 1200 h) measured during 24 h periods over 7 days.

PM Variable	ALT Experimental Feeding	CON Control Feeding	SEM ^a^	*p*-Value
Downwind PM_2.5_ concentration, mg/m^3^	0.072	0.115	0.007	0.004
Upwind PM_2.5_ concentration, mg/m^3^	0.035	0.036	0.006	0.87
Net PM_2.5_ concentration **^b^**, mg/m^3^	0.036	0.080	0.013	0.042

**^a^** SEM = Standard error means. **^b^** Net PM_2.5_ concentration downwind-upwind PM_2.5_ concentration, in mg/m^3^.

**Table 5:** Performance of cattle under two different feeding time regimens (ALT = fed at 0700, 1000, 1830 h vs. CON = 0700, 1000 and 1200 h) over a period of 152 days on feed.

Measure	Mean			
	CON	ALT	SEM ^a^	*p*-Value
Number of pens	7	7	-	-
Number of cattle	1228	1585	-	-
Initial BW, kg	354.9	354.8	1.18	0.93
Final BW, kg	570.9	563.8	3.05	0.14
ADG, kg/day	1.42	1.36	0.02	0.095
ADG, kg/day, with DMI as a covariate **^b^**	1.39	1.39	0.02	0.88
Feed:gain ratio	6.13	6.21	0.10	0.61
DMI, kg/day	8.71	8.40	0.06	0.004

**^a^** SEM = Standard error means. **^b^** These means statistically adjust the raw means as if DMI was identical among CON and ALT cattle.
